# Dissemination of enteroviruses in the production chain of organic lettuce in Rio de Janeiro, Brazil

**DOI:** 10.1002/mbo3.653

**Published:** 2018-08-15

**Authors:** Lucia M. C. Werneck, Marcia Leite Baptista, Marize P. Miagostovich, Edson Elias da Silva

**Affiliations:** ^1^ Oswaldo Cruz Foundation (Fiocruz) ‐ National Institute of Quality Control in Health (INCQS) Rio de Janeiro Brazil; ^2^ Oswaldo Cruz Institute (IOC) Rio de Janeiro Brazil

**Keywords:** agriculture, environmental health, food safety, viruses, water quality

## Abstract

This study aimed to survey the environmental dissemination of enterovirus (EV) in a site of organic lettuce situated in the mountainous region of the state of Rio de Janeiro, Brazil. For this purpose, a total of 96 environmental samples, including water and lettuce samples obtained in different stages of the production chain (e.g., irrigation water, seedlings, lettuces grown, and washed lettuces ready‐to‐eat), were analyzed. EV genomes were detected in 12.5% (12/96) of the tested samples (eight from irrigation water and 4 from lettuce samples). Levels of viral concentration ranged from 3.37 × 10^3^ to 4.72 × 10^6^ genomic copies per liter (gc L^−1^) and from 2.14 × 10^4^ to 5.56 × 10^4^ genome copies per 25 grams (gc 25 g^−1^) for the water and lettuce samples, respectively. Such findings suggest that the use of viruses as human fecal contamination markers must be considered in order to improve food safety in organic supply chains.

## 1 INTRODUCTION

Organic lettuce (*Lactuca sativa*) has been acknowledged as an important transmission source of enteric viruses, since it is traditionally eaten raw or receives minimal processing (Gerba & Choi, 2006). Due to their resistance to adverse conditions, the low infectious dose and the large number of infectious particles, these viruses have been described as important environmental contaminants (De Giglio et al., 2017; Grassi et al., 2010). Among those viruses are the human enteroviruses (EVs), which are associated with asymptomatic infections or mild diseases, such as the common cold or minor undifferentiated febrile illnesses. Yet under certain conditions, EVs also cause serious human diseases such as poliomyelitis, meningitis, encephalitis, myocarditis, and hand, foot, and mouth disease (European Centre for Disease Prevention and Control (ECDC), 2016; Faustini et al., 2006; Khetsuriani, Lamonte‐Fowlkes, Oberst, & Pallansch, 2006; Starlin et al., 2001; Zhu et al., 2007).

Originally EV were classified into four groups: poliovirus (PV), coxsackie A virus (CA), coxsackie B virus (CB), and human orphan cytopathic enteric virus (echovirus), but it was noted that there were significant overlaps in the biological properties of those viruses in different groups. Currently, the *Enterovirus genus*, classified within the *Picornaviridae* family, consists of 13 species including *Enterovirus* A‐J and *Rhinovirus* A‐C, in which the species of *Enterovirus* A, B, C, D and *Rhinovirus* A, B, C, infect humans (http://www.picornaviridae.com/enterovirus/enterovirus.htm; International Committee on Taxonomy of Viruses (ICTV), 2017).

EV was associated with the first transmission of food‐borne viruses reported in 1914, when polio outbreaks were associated with milk consumption (Jubb, 1915). More recently, there are no reports of food‐borne outbreaks associated with EV (Todd and Greig 2015). However, studies have detected EV in several matrices including sewage, irrigation water, and shellfish, some of them from environmental surveillance studies of polioviruses and nonpolio enterovirus (Cheong et al., 2009; Connell et al. 2012; De Oliveira Pereira et al., 2016; Espinosa, Arias, Sánchez‐Colón, & Mazari‐Hiriart, 2009; Ndiaye, Diop, & Diop, 2014). Elsewhere, some studies have shown that EV can be transferred onto the surface of vegetables through spray irrigation water resulting in viral contamination of the vegetables (Allende & Monaghan, 2015; Cheong et al., 2009; Pachepsky, Shelton, McLain, Patel, & Mandrell, 2011; Uyttendaele et al., 2015).

This study aims to investigate the environmental dissemination of human *Enterovirus* species in the production chain of organic lettuce in a small farm located in the region of Brejal, municipality of Petropolis, in the state of Rio de Janeiro, Brazil.

## 2 MATERIALS AND METHODS

### 2.1 Study site, sampling collection, and viral concentration methods

Water and lettuce samples were obtained at different lettuce production stages between August 2010 and August 2011 in an organic production site located in Brejal region, municipality of Petropolis, in the mountains of Rio de Janeiro. Ninety‐six samples including seedling irrigation water (W1), catchment spring water (W2), dam water (W3), lettuce irrigation water (W4), lettuce wash water (W5), and three lettuce samples as seedlings (L1), lettuces grown (L2), and washed lettuce (L3), previously concentrated and investigated for rotavirus, norovirus, and human adenovirus (Werneck et al., 2017) were processed for EV investigation.

Water samples (2 L) were processed for viral concentration using an adsorption‐elution method with a negatively charged membrane as described previously by Katayama, Shimasaki, and Ohgaki (2002). For lettuce samples, 25 grams (g) was concentrated using the same method adapted for small volumes (approximately 120 ml) using a 0.45 μm negative charge membrane with a Stericup^®^ filter (250 ml) (Nihon, Millipore, USA) and ultrafiltration was performed with Centriprep Concentrator^®^ 50 Nihon, Millipore) to give a final volume of 2 ml (Fumian, Leite, Marin, & Miagostovich, 2009; Katayama et al., 2002).

RNA extraction and One‐step reverse transcriptase quantitative polymerase chain reaction (RT‐qPCR).

Nucleic acids were extracted using QIAamp Viral RNA Mini Kits (Qiagen, Inc., Valencia, CA, USA), according to the manufacturer's instructions.

RT‐qPCR for EV was developed and standardized by the National Enterovirus Reference Laboratory. With an aim to improve the detection and quantification of EV by qPCR, primers and probes corresponding to the 5′NC region of human EV were designed and evaluated for species A to D detection (Da Silva, E.E. ‐ Unpublished data). For a qPCR standard curve the RNA from poliovirus Sabin 3 (NIBSC code: 01/532) obtained from human RD cell cultures was extracted and diluted from 10^−1^ to 10^−9^. To determine the limit of detection standard RNA was serially diluted in a pool of negative water concentrates. The concentrates were extracted as described above and tested in duplicate.

RT‐qPCR was performed using AgPath‐ID^™^ One‐step RT‐PCR Kit (Applied Biosystems, California, USA). Nine microliter of RNA extracted was added to the reaction mix according to manufacturer's instructions. Assays were placed into an ABI PRISM 7500 Sequence Detection System (Applied Biosystems, Foster City, CA) on the following conditions: incubation at 50°C for 2 min to activate UNG, initial denaturation at 95°C for 10 min, and then 40 cycles of 95°C for 15 s and 56–60°C for 1 min. Amplifications were analyzed in duplicated, and positive and negative controls were included in each run. Virus concentration results are present as gc L^**−**1^ and gc 25 g^**−**1^ for water and lettuce samples, respectively.

### 2.2 Virus isolation

According World Health Organization (2004), all positive samples were inoculated into three different cell lines sensitive to the isolation of the genus *Enterovirus*: human RD, MRC‐5, and HEp 2c. Cells were supplied by the Center for Disease Control and Prevention (CDC; Atlanta, USA) to the Fiocruz/RJ Enterovirus Laboratory. Briefly, 200 μl of concentrate was inoculated in duplicate into 12 ml cell culture tubes (Nalgene^™^ Thermo Fischer Scientific, USA) containing 1.0 x10^5^ cells/ml. Culture cells were kept at 36°C with daily observation using an invert microscope for the appearance of cytopathic effect. Three serial passages were conducted with a 7‐day interval. Control cultures cells were used to report toxicity, degeneration, or contamination. After 21 days, the qPCR was performed with paired samples (initial concentrates and inoculated cultures). The same procedure was accomplished for the three cell types used.

## 3 RESULTS AND DISCUSSION

This study investigated the presence of human *Enterovirus* species using one‐step reverse transcriptase quantitative TaqMan^®^ PCR (RT‐qPCR) from water and lettuce samples obtained from a chain of organic lettuce. The limit of detection in 100 μl of water of RT‐qPCR assays that were used in this study was found to be 168 gc for EV following the FSA 2006 guidelines (Armbruster & Pry, 2008; FSA 2006). The RT‐qPCR assay detected EV in 12.5% (12/96) of total samples, 13% (8/60) from water, and 11% (4/36) from lettuce samples. EV load ranged from 3.37 × 10^3^ to 4.72 × 10^6^ genomic copies per liter (gc L^**−**1^) and from 2.14 × 10^4^ to 5.56 × 10^4^ genome copies per 25 grams (gc 25 g^**−**1^) for water and lettuce samples, respectively (Table 1). Previously, a study carried out with these same samples revealed high contamination with the main gastroenteric viruses (i.e., rotavirus, norovirus and, adenovirus) with detection of at least one of them in all the samples studied and with the rotavirus concentration reaching 10^4^ gc 25 g^**−**1^ in samples of lettuce ready for consumption (Werneck et al., 2017). A comparative analysis with the previous results shows that the EV were obtained in a higher concentration in the seedling's irrigation waters (10^6^ gc L^**−**1^). However, in the lettuce samples, mean values of EV were detected in concentrations similar to RVA data, and higher than HAdV (10^2^ gc L^**−**1^), which was the best indicator since they were present in at least one sample from each point of the production chain. The detection of viral concentration ranging from 10^2^ to 10^4^ gc 25 g^**−**1^ in adult lettuce suggests that sometimes the level of viral contamination increased at the end of production. However, it is not possible to state whether there is a growing process of contamination for all viruses, mainly due to the heterogeneous distribution of them commonly observed in environmental samples. It was also observed that lettuces washed at the last stage of production do not represent a benefit for virus removal, since the waters used are also contaminated. Enteric viruses including EV have been used as indicators of sanitation and hygiene practices at food production sites on dairy and swine farms (Fongaro et al., 2014; Kokkinos et al., 2012; Lachapelle, Letellier, Fravalo, Brassard, & L'Homme, 2017; Maunula et al., 2013; Staggemeier et al., 2015; Yavarmanesh, Alum, & Abbaszadegan, 2015).

**Table 1:** Detection and quantification of enterovirus (EV) according to matrix and collection points (*n* = 12 each)

Matrix	Sample collection points	Number of positives (%)	EV concentration per genome copies (gc)
Water	W1	4 (33.3)	6.36 × 10^4^ a 7.62 × 10^5^ 3.37 × 10^3^ 4.72 × 10^6^
	W2	2 (16.7)	2.03 × 10^5^ 1.64 × 10^6^
	W3	1 (8.3)	5.14 × 10^3^
	W4	1 (8.3)	2.37 × 10^4^
	W5	ND	ND
Lettuce	L1	ND	ND
	L2	2 (16.7)	3.28 × 10^4^ b 5.47 × 10^4^
	L3	2 (16.7)	2.14 × 10^4^ 5.56 × 10^4^

ND, Not detected.

a Water (gc L^−1^).

b Lettuce (gc 25 g^−1^).

W1. Seedling irrigation water; W2. Catchment of spring water; W3. Dam; W4. Lettuce irrigation water; W5. Lettuce wash water; L1. Seedlings; L2. Dried lettuce; L3. Washed lettuce.

In order to demonstrate EV infectivity, all 12 EV‐positive samples by RT‐qPCR were processed to attempt virus isolation following the World Health Organisation (WHO) protocol described previously (World Health Organization, 2004). No EV characteristic cytopathic effects were observed after three consecutive passages of 7 days each in cell cultures. After this period, RT‐qPCR was carried out for all different culture cell lines in paired samples (initial concentrates and cultures) with no reduction in threshold cycle (Ct) values, revealing no virus replication. Human rhabdomyosarcoma (RD), human diploid cells derived from lung (MRC‐5) and human epithelial carcinoma cells (HEp 2c) are susceptible to EV infection and were used to improve successful isolation as recommended by WHO. This protocol is routinely performed by EV laboratories that usually inoculate specimens into a minimum of three cell lines (World Health Organization, 2004, World Health Organization (WHO), 2015). Although it has not been possible to demonstrate the infectivity of the EVs detected, the presence of the viral genome is sufficient to demonstrate the potential risk of infection from the consumption of those raw products. It was also not possible to demonstrate HAdV infectivity from those same samples, corroborating the idea previously mentioned that a long interval for performing assays may have influenced these results (Werneck et al., 2017).

In conclusion, our results point out the need of considering and monitoring enteric viruses, as environmental contaminants, mainly in food producing areas that do not meet the requirements of adequate sanitation. Worldwide, food security has been a growing concern for authorities that are stepping up efforts in an attempt to minimize harm to health (World Health Organization, 2002; Centers for Disease Control and Prevention (CDC), 2016). In this context, viruses emerge as a challenge since their contamination in the environment is difficult to eliminate, reinforcing the importance of basic sanitation in food security (Cook, Knight, & Richards, 2016).

## CONFLICT OF INTEREST

No conflict of interest declared.
